# Covariation of Cigarette Smoking and Other Health-risk Behaviors among Japanese High School Students: a Preliminary Study

**DOI:** 10.2188/jea.11.224

**Published:** 2007-11-30

**Authors:** Minoru Takakura, Masaru Ueji, Seizo Sakihara

**Affiliations:** 1 School of Health Sciences, University of the Ryukyus.; 2 Institute of Community Medicine, University of Tsukuba.

**Keywords:** cigarette smoking, health-risk behavior, covariation, high school students, adolescents

## Abstract

This study aims to determine if cigarette smoking is associated with engaging in other health-risk behaviors among high school students in Japan. Self-administered anonymous questionnaires were conducted in 1999 using a sample of 1,466 students (male: 50.5%, female: 49.5%) in grades 10 through 12 at seven public senior high schools in urban areas of Okinawa, Japan. Health-risk behaviors studiedal included cigarette smoking, alcohol drinking, thinner use, sexual intercourse, suicidal ideation, nonuse of seat belts, physical inactivity, and weight loss practices. In the logistic regression models, controlled for sociodemographic variables, smoking was significantly associated with all health-risk behaviors except physical inactivity. In particular, associations of alcohol drinking and sexual intercourse with smoking were strong. Among male students, statistically significant odds ratios existed for alcohol drinking, sexual intercourse, and nonuse of seat belts. Among female students, all of the odds ratios for health-risk behaviors were statistically significant, except for physical inactivity. Generally, the odds ratios of female students were higher than those of male students. In conclusion, high school students who smoked cigarettes in this study may be at higher risk for engaging in other health-risk behaviors. Particularly, alcohol drinking and sexual intercourse are more likely to co-occur with smoking. These findings suggest that smoking prevention programs should be integrated with other health-risk behaviors.

## INTRODUCTION

Cigarette smoking is the leading preventable cause of premature death in Japan as well as in the United States^1^^, ^^2^^)^. The prevalence of smoking among Japanese male adults remains higher rate in the developed countries. Recently, smoking rate has been also increasing among young female adults and adolescents^3^^)^. The nationwide surveys of Japanese adolescent smoking indicated that the current smoking rate among the 12th graders in 1990 was 26% for males and 5% for females, and it increased to 37% and 16% respectively in 1996^4^^, ^^5^^)^. Based on current trend, adolescent smoking will remain a major health problem in Japan.

Cigarette smoking among adolescents has been found to predict use of alcohol and other drugs and to act as a so-called “gateway drug.”^6^^)^ Therefore, adolescent smoking may serve as a warning signal of the presence of other drugs’ use. On the other hand, adolescent health-risk behaviors that are also problem behaviors, such as drug use, alcohol abuse, delinquency, and sexual precocity, are likely to cluster or accumulate^7^^, ^^8^^)^. Many studies on adolescent smoking have also indicated a high covariation with these problem behaviors. For instance, adolescents who reported current smoking were more likely to engage in intentional injury risk behaviors and sexual behaviors as well as illicit drug use^9^^-^^11^^)^. Similarly, cigarette smoking was related to non-problem, health-related behaviors, such as eating, physical activity, and safety behaviors^12^^-^^14^^)^.

Studies of covariations of smoking and other health-risk behaviors may provide opportunities to identity high-risk adolescents who accumulate health-risk behaviors and have important practical implications with regard to the development of intervention programs. However, available data are not sufficient about which health-risk behaviors may co-occur with smoking in Japanese adolescents because previous studies in Japan tended to concentrate on pairwise associations between smoking and use of other drugs, such as alcohol and thinner^15^^, ^^16^^)^.

This study aims to determine if cigarette smoking is associated with engaging in other health-risk behaviors among Japanese high school students.

## METHODS

### Procedures and Subjects

Using written instructions provided by researchers, classroom teachers conducted a self-administered anonymous questionnaire survey in classroom settings in September 1999. After being informed of the nature and intent of the study both in writing and verbally, the students were requested to complete the questionnaire and return it sealed in the return envelope to assure the validity of the responses and confidentiality. Students who did not want to participate in the study could decline to respond and designate that on the front sheet of the questionnaire. Students who were absent from school when the survey was conducted were not followed up.

The study sample consisted of 1,466 students enrolled in one to two classes in grades 10 through 12 (ages 15-18) at seven public senior high schools in urban areas of Okinawa, Japan. These schools were chosen from four general high schools and three vocational high schools, based upon willingness of school administrators to participate in the study. The questionnaires were collected from 1,317 students. Twelve students declined to participate and 137 students were absent from school when the survey was conducted. A total of 1,290 students completely responded in smoking status (630 males, 660 females), and their data were used in analysis.

### Measures

We selected eight questions dealing with health-risk behaviors that took into consideration leading causes of mortality and morbidity and current health trends in Japan. These questions were measured by using standard items adapted mainly from the Youth Risk Behavior Surveillance (YRBS) conducted by the US Centers for Disease Control and Prevention (CDC)^17^^)^. These questions were translated into Japanese and were reviewed for content validity by school principals, school teachers, school nurses, and researchers. No revisions to the appearance of the questions were necessary. Test-retest reliabilities of several questions for Japanese high school students were determined^18^^)^. The students were asked the following questions about tobacco use, alcohol and other drug use: “During the past month, how many cigarettes did you smoke per day? ”, “During the past month, on how many days did you drink alcohol? ”, and “During your life, have you ever inhaled thinner? ” A current smoker or drinker was defined as someone who had smoked at least one cigarette or consumed an alcohol beverage one day in the past month. Respondents who had the experience of inhaling thinner were also considered solvent users. To evaluate behaviors related to injuries, the students were asked, “How often do you wear a seat belt when riding in a car driven by someone else? ” and “During the past 12 months, did you ever seriously consider attempting suicide?” Respondents who never or rarely wore seat belts were categorized as nonusers. Suicidal risk adolescents were persons who reported serious suicidal ideation. To evaluate sexual behaviors, the students were asked, “During your life, have you ever had sexual intercourse? ” Respondents who said “yes” were categorized as having had sexual intercourse. The following question about dieting behaviors was asked: “During the past 12 months, did you do any of the following to lose weight: vomit or take diet pills? ” Weight-loss attempters were persons who had practiced one of those behaviors. The students were also asked, “On how many of the past 7 days, did you participate in activities that made you sweat and breathe hard for at least 20 minutes? ” Physically inactive adolescents were persons who participated in vigorous physical activity on two or less days in the past week. Moreover, sociodemographic variables including gender, grade level, school type, and parental education level were assessed.

### Data analysis

To determine odds ratios (ORs) and confidence intervals (CIs) for health-risk behaviors among current smokers as compared with non-smokers, logistic regression analyses for total sample, controlled for gender, grade level, school type, and parental education, were conducted. To examine gender differences, separate logistic regression analyses for males and females, controlled for grade level, school type, and parental education, were conducted.

## RESULTS

Current smoking status of high school students by sociodemographic characteristics is shown in Table 1. Overall, 17.4% of the students smoked cigarettes. The prevalence of smoking was significantly higher among male students, vocational high school students, and those whose parental education level was less than high school.

**Table 1.** Prevalence of current smoking status by sociodemographic characteristics.

Characteristic		Non-smokers		Smokers		*χ* ^2^	p
	
		n	%	n	%		
Grade							
	10th	388	(84.3)	72	(15.7)	2.996	0.224
	11th	389	(83.1)	79	(16.9)		
	12th	289	(79.8)	73	(20.2)		
Gender							
	Male	467	(74.1)	163	(25.9)	62.128	<0.001
	Female	599	(90.8)	61	(9.2)		
School type							
	General	744	(91.7)	67	(8.3)	126.128	<0.001
	Vocational	322	(67.2)	157	(32.8)		
Parental education							
	≦High School	512	(79.6)	131	(20.4)	9.574	0.008
	≧University*	481	(86.4)	76	(13.6)		
	Unknown	73	(81.1)	17	(18.9)		

Total		1066	(82.6)	224	(17.4)		

* Including junior college graduates.

Table 2 shows the prevalence of health-risk behaviors by smoking status among high school students. All of health-risk behaviors were significantly more prevalent among current smokers than among non-smokers, except for physical inactivity in total sample and females, and nonuse of seat belts in females.

**Table 2.** Percentages of the students engaging in health-risk behaviors by current smoking status.

	Current smoking status					

	Total		Males		Females	
		
	No	Yes	No	Yes	No	Yes
Current drinking	30.2	70.5	31.1	68.1	29.6	76.3
Thinner use	2.0	8.0	3.4	7.0	1.0	10.5
Sexual intercourse	13.2	46.7	12.0	40.8	14.1	61.3
Suicidal ideation	8.3	17.6	6.6	12.4	9.6	30.3
Nonuse of seatbelts	27.0	41.3	25.7	42.6	28.0	38.2
Physical inactivity	40.8	44.2	27.3	36.8	51.0	61.8
Weight loss	2.4	6.6	0.0	1.1	4.3	20.0

All *χ*^2^ tests showed p<0.05, except for physical inactivity in total and females and nonuse of seatbelts in females (p≧0.05).

The ORs and CIs for health-risk behaviors among the students who smoked cigarettes as compared with those who did not smoke are shown in Table 3. All of the ORs were greater than 1.0. Overall, smoking was significantly associated with all health-risk behaviors except physical inactivity. In particular, associations of alcohol drinking and sexual intercourse with smoking were strong. As for gender differences, statistically significant ORs existed for alcohol drinking, sexual intercourse, and nonuse of seat belts among male students. Among female students, all of the ORs for health-risk behaviors were statistically significant, except for physical inactivity. Generally, the ORs of female students were larger than those of male students were.

**Table 3.** The odds ratios for health-risk behaviors among current smokers as compared with non-smokers.

	Total		Males		Females	
		
	**OR^†^**	95% CI^¶^	OR^‡^	95% CI^¶^	OR^‡^	95% CI^¶^
Current drinking	**4.9**	(3.5 , 6.9)	**4.8**	(3.2 , 7.3)	**6.3**	(3.3 , 11.8)
Thinner use	**3.0**	(1.5 , 6.0)	1.3	(0.6 , 3.2)	**15.3**	(4.7 , 49.5)
Sexual intercourse	**5.8**	(4.1 , 8.3)	**4.7**	(3.0 , 7.4)	**10.1**	(5.4 , 18.9)
Suicidal ideation	**2.0**	(1.3 , 3.2)	1.5	(0.8 , 2.9)	**3.1**	(1.6 , 5.9)
Nonuse of seatbelts	**2.2**	(1.6 , 3.0)	**2.1**	(1.4 , 3.1)	**2.1**	(1.2 , 3.7)
Physical inactivity	1.1	(0.8 , 1.6)	1.1	(0.7 , 1.7)	1.2	(0.7 , 2.1)
Weight loss	**2.6**	(1.2 , 5.9)	**–**	–	**3.2**	(1.4 , 7.1)

Bolded odds ratios indicate a statistically significance.

^†^ Adjusted for gender, grade, school type, and parental education.

^‡^ Adjusted for grade, school type, and parental education.

^¶^ 95% Confidence Interval.

Ellipsis indicates data not available.

## DISCUSSION

In this study, after adjustment for the influence of sociodemographic variables, high school students who smoked cigarettes were more likely to engage in most of other health-risk behaviors. Previous studies reported that frequency of cigarette smoking was positively related to the prevalence of substance use such as alcohol, marijuana, cocaine, and thinner^6^^, ^^9^^, ^^10^^, ^^15^^)^. Other studies found that adolescents who reported cigarette smoking also reported higher rates of health risk and problem behaviors, including physical fighting, weapon carrying, suicide attempts, nonuse of seat belts, sexual behaviors, and unhealthy weight loss behaviors^9^^, ^^10^^, ^^13^^, ^^14^^)^. Therefore, the present findings were consistent with the results of previous studies that showed a positive association between smoking and other health-risk behaviors among adolescent populations.

This study also showed that, overall, alcohol drinking and sexual intercourse were strongly correlated with current smoking. Furthermore, among female students, this trend appeared to be particularly strong, and thinner use was found the strongest association with smoking. The evidence for covariation has been strongest for problem behaviors such as substance abuse, delinquency, and sexual precocity, namely, problem behavior syndrome^19^^)^. A common underlying factor that reflects a tendency toward unconventionality is thought to be common to these problem behaviors^20^^)^. Our earlier study^21^^)^ reported that cigarette smoking, alcohol drinking, and sexual intercourse were interrelated and proposed that there was an accumulation of these three risk behaviors. Thus, our findings may suggest that cigarette smoking, alcohol drinking, and sexual intercourse are more likely to co-occur as a part of problem behavior syndrome among Japanese adolescents.

It was interesting that the ORs of female students were higher than those of male students were, which indicated that the covariations of cigarette smoking and other health-risk behaviors were more apparent in female students. Therefore, female smokers may increase vulnerability to engaging in health-risk behaviors compared with male smokers, and they may represent a high-risk group among Japanese adolescents. The reason for the different association among males and females is likely due to low smoking rate in female students. It is also possible that cigarette smoking among females is rather problematic and thus is highly correlated with other problematic behaviors, while cigarette smoking among males is more prevalent and common. Additionally, Takakura et al^22^^)^ indicated that as the number of risk factors increased, magnitude of risk for smoking among females became considerably great compared with those of males. In this study, female smokers who reported other health-risk behaviors too may be extremely exposed to many risk factors.

Some previous studies have reported that physical inactivity was significantly associated with smoking among adolescents^12^^, ^^23^^)^. However, this study showed no significant association between these two behaviors among both male and female students. Similarly, Pesa^14^^)^, using a national sample of Mexican-American adolescents, found no significant association between smoking and sports participation among boys and explained that there may be a sanctioning effect within the sports environment for involvement in unhealthy behaviors. On the other hand, in Japanese students, physical inactivity may be mostly remedied by participation in physical education classes. Therefore, physical inactivity occurred independently among Japanese high school students.

Jessor^19^^)^ has developed a comprehensive model for explaining adolescent risk behavior, which incorporated five explanatory domains such as social environment, perceived environment, personality, behavior, and biology/genetics. These five domains would constitute a complexity of the web of causation. He also mentioned “what that complexity suggests is that prevention and intervention efforts that are comprehensive promise to yield greater success than those that are more limited in scope.” Thus, prevention programs directed at the patterning of multiple risk behaviors may be more appropriate than programs focused on specific behavior alone. Besides, it was hypothesized that those behaviors strongly associated with each other shared common etiologic or underlying factors and those weakly associated with each other had mainly unique underlying factors^24^^)^. Therefore, the results of this study suggest that smoking prevention intervention should take into consideration integration with other health-risk behaviors such as alcohol drinking and sexual intercourse in particular. It should be emphasized the importance of focusing on common underlying factors leading to the clustering of health-risk behaviors. Additionally, these findings also suggest that if adolescent cigarette smoking is occurring, further screening and counseling should not be limited to smoking issues only but should be more comprehensive.

One limitation of this study is our sample selection process as this study was carried out exclusively on public high school students in Okinawa Prefecture and those in schools had principals who agreed to participate in the survey. Moreover, these data apply only to students who attended high school. Therefore, the present findings must be interpreted cautiously and limited to generalize to adolescents in Japan as a whole. Another limitation is that all health-risk behaviors were assessed by only one question each. It is not clear therefore whether each question most effectively measured each of the aiming health-risk behavior. In these circumstances, this study should be considered a preliminary study of the association between smoking and health-risk behaviors among Japanese adolescents. Although random sampling data are very difficult to obtain in Japanese school settings, further research is strongly needed based on various samples of adolescents in Japan as a whole and using more reliable and valid measures of health-risk behaviors.
